# The relationship of early recurrence of atrial fibrillation and the 3-month integrity of the ablation lesion set

**DOI:** 10.1038/s41598-018-28072-y

**Published:** 2018-06-29

**Authors:** Nebojša Mujović, Milan Marinković, Nebojša Marković, Vera Vučićević, Gregory Y. H. Lip, T. Jared Bunch, Tatjana S. Potpara

**Affiliations:** 1 Cardiology Clinic, Clinical Center of Serbia, Belgrade, Serbia; 2 School of Medicine, University of Belgrade, Belgrade, Serbia; 3 Center for Anesthesiology and Reanimatology, Clinical Center of Serbia, Belgrade, Serbia; 4 Cardiology Department, City Hospital, Birmingham, United Kingdom; 5 Institute of Cardiovascular Sciences, University of Birmingham, Birmingham, United Kingdom; 6 Intermountain Medical Center Heart Institute, Intermountain Medical Center, Murray, Utah United States; 7 Stanford University, Department of Internal Medicine, Palo Alto, California United States

## Abstract

Early recurrence of atrial fibrillation (ERAF) after catheter-ablation (CA) can be a transient phenomenon due to inflammation, or a harbinger of late AF recurrence due to CA lesion (re)conduction. We studied the relationship between ERAF and the 3-month CA lesions integrity. Forty one consecutive AF patients who underwent a pulmonary vein isolation (PVI), roof line (RL) and mitral isthmus line (MIL) CA were enrolled. At 3 months all patients underwent invasive assessment of the lesion set integrity irrespective of ERAF. The PVI, RL and MIL ablation was successful in 100.0%, 95.1% and 82.9% patients, respectively. At the 3-month remapping, a gap in PVI-lesion(s), RL or MIL was identified in 61.0%, 31.7% and 36.6% patients, respectively. Patients with (n = 17, 41.5%) compared to those without ERAF (n = 24) had a significantly higher rate of any PV-reconnection (88.2% vs. 41.7%), the right PV(s)-reconnection (82.5% vs. 29.2%) and the RL gap (52.9% vs. 16.7%), as well as a higher number of reconnected right PVI-segments, all p < 0.05. On multivariate analysis, only the number of reconnected right PVI-segments was associated with ERAF (OR 4.26, p = 0.004). The ERAF following PVI + RL + MIL ablation was significantly related to 3-month PV-reconnections and the presence of RL gaps.

## Introduction

The incidence of early recurrence of atrial fibrillation (ERAF) after AF catheter-ablation (CA) is approximately 40%^1^. Sometimes ERAF is only a transient phenomenon attributed to the ablation-related inflammation and/or autonomic denervation^1–3^. Hence, observance of a 3-month “blanking” period is advocated prior to the definite CA outcome assessment^1–3^.

Nevertheless, ERAF is one of the strongest predictors of late recurrence of atrial arrhythmias and can be related to conduction gaps in the ablation lesion(s)^3–6^. Identifying the higher risk sites for gaps may allow upfront modification of routine ablation strategies such as increasing energy delivery to improve the likelihood of durable lesion at these critical sites in order to reduce the incidence of ERAF and the late arrhythmia recurrence.

To date, the relationship between ERAF and the long-term integrity of an ablation lesion set is underreported. Only one study of paroxysmal AF patients has invasively assessed the relationship between the durability of pulmonary vein isolation (PVI) and ERAF and found that after 4 weeks ERAF was associated with both PV reconnection and the number of veins with gaps^5^.

However, the prognostic significance of ERAF depends on ablation strategy as additional substrate-based ablation on top of PVI may further impact degree of inflammation and autonomic disruption and increase the proportion of transient ERAF cases that are not associated with late arrhythmia recurrence^6^. We have recently reported on the persistency of the extensive left atrial (LA) lesion set (i.e., PVI combined with the roof line [RL] and the mitral isthmus line [MIL]) at the 3-month follow-up invasive electrophysiological study^7^. In the present study we examined the relationship between ERAF and the integrity of the PVI + RL + MIL lesion set created during the index CA of AF, as assessed at 3 months in consecutive patients who all underwent the identical index CA procedure.

## Methodology

### Study design

This prospective study was conducted in the Clinical Center of Serbia. All participants underwent the two invasive procedures: (1) the index CA of AF, which included a circumferential PVI accompanied with the RL and MIL ablation, and (2) the follow-up electrophysiological study after the 3-month “blanking” period post index CA, irrespective of arrhythmia outcome, to evaluate the integrity of the index ablation lesions and, if necessary, to re-ablate residual conduction gaps. The study protocol was approved by the hospital ethics committee (Clinical Center of Serbia, Ethics committee approval #1860/21, 8^th^ October 2015). All participants gave written informed consent to undergo both invasive procedures.

### Patient selection

Between November 2015 and February 2016 a total of 65 patients underwent CA of AF in our center. Of these, 41 consecutive patients underwent their index CA of symptomatic drug-refractory AF, using the pre-specified set of ablation lesions (PVI + RL + MIL) and were enrolled in the present study. In our center, this lesion set is used in patients with persistent AF and in patients with paroxysmal AF if their AF episodes have lasted >48 hours reflecting more complex LA substrate^3,8^. Patients were excluded from the present study if they had a history of previous CA of AF (n = 8), or their ablation consisted of stand-alone PVI (n = 13) or included an alternative strategy for substrate modification (n = 3).

The scheduled 3-month invasive follow-up electrophysiological study was performed between March and May 2016. After the follow-up procedure all participants were clinically followed until July 2017.

All patients were appropriately anti-coagulated for ≥6 weeks before the CA^2,3^. All anti-arrhythmic drugs (AADs) were ceased >5 half-lives prior to CA, except for amiodarone which was discontinued 3 months before the procedure.

### Index CA

The strategy for CA using the above mentioned set of lesions and the electro-anatomical mapping system (EnSite Velocity, St Jude, Minneapolis MN) was described in detail recently^7^. All invasive procedures were performed under a deep sedation with propofol, midazolam and fentanyl. In patients with ongoing AF or atrial tachycardia (AT) at the beginning of the procedure sinus rhythm was restored by electrical cardioversion. However, before cardioversion, in patients with regular AT or atrial flutter a tachycardia circuit was determined by activation and entrainment mapping.

The order of the LA ablation targets during the procedure was as follows: left PVs, right PVs, the RL and the MIL. The recovery of conduction across the ablated lesion (PVs, the RL or MIL) recorded during the procedure was considered as the acute reconnection and re-ablation was attempted.

### PVI

**Figure 1:**
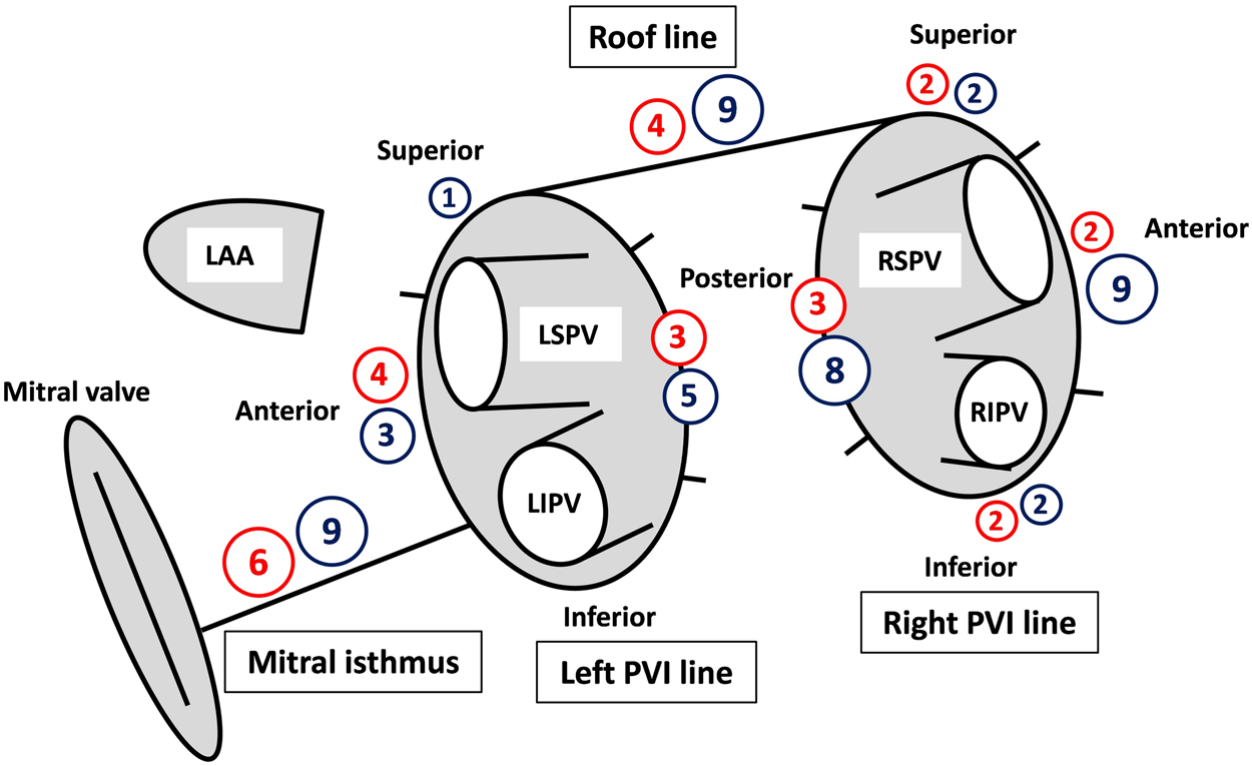
Schematic illustration of the pre-specified ablation lesion set and the distribution of 3-month conduction gaps. The encircling PVI lesions were arbitrarily divided in 4 anatomical segments: superior, anterior, inferior and posterior. The 3-month conduction gaps in patients with and without ERAF were represented with dark blue and red circles, respectively. The absolute number of gaps in each segment was given within the circle and size of the circle roughly correlates with the number of local gaps. LAA, left atrial appendage; PVI, pulmonary vein isolation; LSPV, left superior pulmonary vein; LIPV, left inferior pulmonary vein; RSPV, right superior pulmonary vein; RIPV, right inferior pulmonary vein.

Navigation of the externally irrigated 4-mm tip ablation catheter (Therapy Cool-Flex, St. Jude Medical) was achieved by deflectable long sheath (Agilis NxT, St. Jude Medical). PV activity throughout the procedure was assessed using a duodecapolar 20 mm diameter circular mapping catheter. PVs were isolated by continuous lesions encircling the ipsilateral PVs, 10–20 mm from the PV ostia^7^. Each of the encircling lesions was divided into the 4 anatomical segments: superior, anterior, inferior and posterior, Fig. 1. The set-up for radiofrequency (RF) ablation at the same site was 43 °C, flow-rate 17 ml/min and 30 W for 40–60 sec on the anterior LA wall and 25 W for 30–40 sec on the posterior LA wall. Criteria used for PVI diagnosis are reported elsewhere^7,9^. After the ipsilateral PVI, an additional ablation at antral segments was continued, if necessary, to achieve “loss-of-pace-capture” (with 10 mA) along the entire PVI line^9^.

### Substrate-based ablation

The RL and MIL was ablated during continues LA appendage pacing from a circular mapping catheter^7,10,11^. Ablation at each site lasted until electrogram amplitude decreases by >80% or potential splits, or to maximally 60–120 sec. The MIL ablation started endocardially, but if necessary, proceeded within the distal coronary sinus (18–25 W for 40–60 sec). The ablation end-point was a conduction block across the line(s) confirmed by standard electrophysiological criteria and is described in detail in our recent report^7^. Ablation of cavo-tricuspid isthmus was performed if typical atrial flutter was documented^2,3,7^.

### Blanking period after the index procedure

The 3-month blanking period was used, as recommended^1–3^. At discharge, the AAD used before the index CA was reintroduced and the same AAD was used, if possible, during the entire blanking period irrespective of arrhythmia recurrence. Afterwards, all AADs were withdrawn. In patients who required amiodarone therapy after the index ablation, the drug was suspended 1 month before the follow-up procedure. Post-procedural follow-up visits with a 12-lead ECG and 24-hour Holter-recording were conducted at discharge, at the months 1, 3, and 6, and thereafter every 6 months^2,3^. Outside the follow-up visits, patients were instructed to obtain an ECG in case of symptoms suggestive of arrhythmia recurrence. In symptomatic patients, additional unscheduled tests were obtained as needed, including 24-hour, 48-hour and/or 7-day Holter-monitoring, spiroergometry, event recorder use for two weeks, and/or intra-hospital telemetry-monitoring. The “ERAF” was defined as the finding of any atrial tachyarrhythmia (AF, AT or atrial flutter) lasting >30 sec during the 3-month blanking period^1–4^. In case of ERAF, pharmacological or electrical cardioversion was performed. Occurrence of these arrhythmias after 3 months post-ablation was considered the “late arrhythmia recurrence”^1–4^.

### Follow-up invasive procedure

At the end of the 3-month blanking period all study participants underwent invasive electrophysiological study using the same protocol and catheter set-up as in the index procedure. Residual PV activity was assessed with circular mapping catheter in sinus rhythm while completeness of the lines was evaluated by pacing maneuvers^7–11^. The “3-month conduction gap” refers to any residual conduction across the previous lesions (due to late reconnection or due to failed index ablation) whilst the “3-month reconnection” was defined more specifically as the resumption of conduction (after successful index ablation). The PVI segments with reconnection were classified anatomically according to position of the earliest PV potential at the circular mapping catheter, see Fig. 1. All identified conduction gaps were re-ablated.

### Statistical analysis

Continuous variables with normal and asymmetrical distribution are presented as mean (±standard deviation, SD) and median with interquartile range (IQR: 25^th^–75^th^ percentile), respectively. Categorical variables are summarized as percentages. All variables were initially analyzed using univariate logistic regression and those with p < 0.10 were included in multivariate analysis. The ERAF-free survival rates were compared using the Kaplan-Meier analysis and the Log-Rank test. Potential relation between the ERAF timing and the 3-month lesion(s) integrity was assessed by the receiving operator curve (ROC) analysis. A two-sided P-value of <0.05 was considered statistically significant. Analyses were performed using the SPSS 18.0 software.

### Data availability

All data generated and/or analyzed during the current study are available from the corresponding author upon reasonable request.

### Ethics approval

The study protocol was approved by the hospital ethics committee (Clinical Center of Serbia, Ethics committee approval #1860/21, 8^th^ October 2015). All participants gave written informed consent to undergo both invasive procedures.

All methods and measurements were performed in accordance with the relevant guidelines and regulations.

## Results

Of 41 patients (mean age 59.7 ± 8.3 years), 78.0% were male and 43.9% had persistent AF. Baseline patient characteristics are presented in Table 1.

**Table 1:** Baseline clinical characteristics of the patients and the index ablation data.

	Total (n = 41)	ERAF (n = 17)	No ERAF (n = 24)	Univariate p
***Baseline clinical data***				
Age (years)	59.7 ± 8.3	62.2 ± 7.1	57.8 ± 8.8	0.102
Male gender	32 (78.0%)	14 (82.4%)	18 (75.0%)	0.577
BMI (kg/m^2^)	28.5 ± 4.6	28.8 ± 4.7	28.3 ± 4.6	0.709
Years since AF diagnosis	5.0 (2.0–10.0)	7.0 (5.0–12.0)	3.5 (1.0–8.0)	0.065
Non-paroxysmal AF	18 (43.9%)	7 (41.2%)	11 (45.8%)	0.767
LV EDD (cm)	53.1 ± 4.7	53.7 ± 4.6	52.7 ± 4.8	0.509
LV EF (%)	60.0 (55.0–65.0)	60.0 (55.0–65.0)	60.0 (55.0–65.0)	0.936
LA dimension (cm)	43.0 ± 5.7	45.0 ± 5.5	41.5 ± 5.5	0.064
CHA_2_DS_2_-VASc score	2.0 (1.0–3.0)	2.0 (1.0–3.0)	1.0 (1.0–2.0)	0.260
Coronary disease	3 (7.3%)	1 (5.9%)	2 (8.3%)	0.768
Cardiomyopathy	5 (12.2%)	2 (11.8%)	3 (12.5%)	0.943
Diabetes	6 (14.6%)	3 (17.6%)	3 (12.5%)	0.647
Hypertension	24 (58.5%)	12 (70.6%)	12 (50.0%)	0.192
CVA	1 (2.4%)	1 (5.9%)	0 (0.0%)	—
Failed AADs	2.0 (1.5–2.5)	2.0 (2.0–2.5)	2.0 (1.0–2.5)	0.511
***The index CA***				
Left PVI line (cm)	11.5 ± 2.1	11.8 ± 1.5	11.2 ± 2.5	0.534
Right PVI line (cm)	12.0 ± 1.8	12.5 ± 1.9	11.7 ± 1.6	0.166
Roof line (cm)	3.0 ± 0.7	3.0 ± 0.7	2.9 ± 0.7	0.815
Mitral isthmus line (cm)	3.6 (3.1–4.0)	3.4 (2.9–3.9)	3.7 (3.2–4.0)	0.317
Procedure time (min)	223.4 ± 46.7	239.1 ± 52.2	212.3 ± 39.8	0.078
RF time (min)	74.1 ± 21.7	84.3 ± 26.4	66.8 ± 14.1	0.024
RF deliveries	52.0 (45.0–67.0)	62.0 (52.0–71.0)	50.0 (44.0–58.5)	0.054
Fluoroscopy (min)	30.8 ± 9.5	31.7 ± 9.1	30.2 ± 9.9	0.627
PV isolation	41 (100.0%)	17 (100.0%)	24 (100.0%)	—
Isolation of the Left PVs “in pair”	27 (65.9%)	10 (58.8%)	17 (70.8%)	0.426
Isolation of the Right PVs “in pair”	13 (31.7%)	4 (23.5%)	9 (37.5%)	0.347
Roof line block	39 (95.1%)	16 (94.1%)	23 (95.8%)	0.803
Mitral isthmus block	34 (82.9%)	15 (88.2%)	19 (79.2%)	0.453
Acute PV(s) reconnection	22 (53.7%)	10 (58.8%)	12 (50.0%)	0.577
Left PV(s) acute reconnection	11 (26.8%)	7 (41.1%)	4 (16.7%)	0.216
Right PV(s) acute	15 (36.6%)	5 (29.4%)	10 (41.7%)	0.330
PVs with acute reconnection (n)	1.0 (0.0–2.0)	1.0 (0.0–2.0)	0.5 (0.0–1.0)	0.397
Typical AFL ablation	5 (12.2%)	2 (11.8%)	3 (12.5%)	0.943

Data are presented as mean ± standard deviation, median (25th to 75th percentile) or numbers (percentages).

ERAF, early recurrence of atrial fibrillation; BMI, body mass index; AF, atrial fibrillation; LV, left ventricle; EDD, end-diastolic dimension; EF, ejection fraction; LA, left atrium; CVA, cerebro-vascular accident; AAD, anti-arrhythmic drug; CA, catheter-ablation; PVI, pulmonary vein isolation; RF, radiofrequency; AFL, atrial flutter.

### The index CA

Successful PVI and block across the RL and MIL at the end of the procedure was achieved in 41 (100%), 39 (95.1%) and 34 patients (82.9%), respectively (Table 1). An acute PV, RL or MIL reconnection during the procedure occurred in 22 of 41 (53.7%), 5 of 39 (12.8%) and 4 of 34 patients (11.8%) with the initially successful lesion, respectively. There was no significant difference in the acute reconnection rate for left and right PVs (11/41 [26.8%] vs. 15/41 [36.6%], p = 0.477). Cavo-tricuspid isthmus ablation was performed in 5 patients (successful in all). Cardiac tamponade developed in 2 patients due to mitral isthmus perforation, which did not interfere with completion of the ablation lesion set and was resolved by pericardiocenthesis (n = 1) or urgent surgery (n = 1).

### ERAF after the index CA

ERAF was documented in 17 of 41 patients (41.6%), mostly in the form of AT (n = 11; 64.7%) or AF (n = 5; 29.4%) and rarely as a typical atrial flutter (n = 1; 5.9%). The arrhythmia was paroxysmal in 11 and persistent in 6 patients. ERAF was diagnosed mostly by symptom triggered ECG (n = 13) or scheduled Holter-monitoring (n = 3), whilst in one symptomatic patient the recurrence was confirmed by additional unscheduled 24-h Holter-monitoring. A single ERAF was documented in 9 patients, and recurrent ERAF in 8 (mean 10.1 ± 8.9 episodes per patient). The earliest ERAF was recorded in the weeks 1-2, 3-4 or 5-6 post-ablation in 11 (64.7%), 4 (23.5%) and 2 patients (11.8%), respectively. The distribution of AADs used throughout the blanking period was similar among the patients with and without ERAF (Table 2). The AAD was changed (verapamil for amiodarone) only in one patient experiencing ERAF. No significant association was found between the time of first ERAF occurrence (in days) and the prevalence of PV reconnections or the gap in the RL and/or MIL (Area Under the Curve: 0.867 [95% CI: 0.68–1.00], p = 0.101) at 3 months.

**Table 2:** Data after the index ablation and from the 3-month electrophysiology procedure.

	Total (n = 41)	ERAF (n = 17)	No ERAF (n = 24)	Univariate p
***AADs at discharge after the index CA***				
Propafenone	21 (51.2%)	9 (52.9%)	12 (50.0%)	0.853
Amiodarone	8 (19.5%)	4 (23.5%)	4 (16.7%)	0.586
Sotalol	8 (19.5%)	3 (17.6%)	5 (20.8%)	0.800
Beta-blockers	3 (7.3%)	0 (0%)	3 (12.5%)	0.999
Verapamil	1 (2.4%)	1 (5.9%)	0 (0%)	>0.999
***Inflammatory markers after the index CA***				
Leukocytes (n, 10^9^/l)	9.0 (7.4–10.7)	8.0 (6.9–10.8)	9.0 (8.0–10.5)	0.843
Neutrophiles (n, 10^9^/l)	6.0 (5.0–8.0)	6.0 (4.9–7.8)	6.0 (5.3–8.5)	0.886
ESR (mm/h)	12.0 (8.0–22.0)	10.0 (6.0–16.0)	13.0 (10.0–26.0)	0.074
Fibrinogen (g/l)	3.5 ± 0.9	3.3 ± 1.0	3.7 ± 0.9	0.249
CRP (mg/l)	12.1 (7.4–19.0)	12.6 (8.0–22.0)	11.5 (6.6–16.0)	0.222
Troponin I (ng/l)	6.0 (5.0–9.0)	5.0 (3.6–9.5)	6.5 (6.0–9.0)	0.719
CK-MB (u/l)	19.0 (16.5–23.5)	19.0 (15.0–22.0)	21.0 (18.0–25.0)	0.287
***3-month Follow-up EP procedure***				
PV reconnection	25 (61.0%)	15 (88.2%)	10 (41.7%)	0.006
Left PVs reconnection	15 (36.6%)	8 (47.1%)	7 (29.2%)	0.245
Right PVs reconnection	21 (51.2%)	14 (82.4%)	7 (29.2%)	0.002
PVs with late reconnection (n)	1.0 (0.0–2.0)	2.0 (1.0–4.0)	0.0 (0.0–2.0)	0.007
Left PVI segments with reconnection (n)	0.0 (0.0–1.0)	0.0 (0.0–1.0)	0.0 (0.0–1.0)	0.170
Right PVI segments with reconnection (n)	1.0 (0.0–1.0)	1.0 (1.0–2.0)	0.0 (0.0–1.0)	0.002
Gap in the Roof line	13 (31.7%)	9 (52.9%)	4 (16.7%)	0.018
Gap in the Mitral isthmus line	15 (36.6%)	6 (35.3%)	9 (37.5%)	0.885

Data are presented as mean ± standard deviation, median (25th to 75th percentile) or numbers (percentages).

ERAF, early recurrence of atrial fibrillation; AAD, anti-arrhythmic drug; CA, catheter-ablation; ESR, erythrocyte sedimentation rate; CRP, C-reactive protein; CK-MB, creatin-kinase MB iso-enzyme; EP, electrophysiology; PV, pulmonary vein; PVI, pulmonary vein isolation.

### Follow-up electrophysiological study

The 3-month electrophysiological study started during macro-reentrant AT in 5 patients: roof flutter (n = 3), right PVI gap-related AT (n = 1) and AT from anterior LA wall (n = 1). A gap in the PVI line(s), RL or MIL was recorded in 5, 4 and 2 of these patients, respectively.

Overall, the 3-month endurance of all lesions was confirmed in 9 of 32 patients (28%) with successful index ablation of all targeted lesions (i.e., PVs, RL, MIL). The distribution of the segments along the lesion sets exhibiting the 3-month gap in patients with and without ERAF is illustrated in Fig. 1.

The 3-month reconnection of any PVs was identified in 25 of 41 patients (61.0%). No significant difference was recorded in the reconnection rate of the left and right PVs (15/41 [36.6%] vs. 21/41 [51.2%], p = 0.266), Table 2. However, the number of PVI segments with the reconnection was significantly higher along the right compared to the left PVI lesion (30/164 [18.3%] vs. 16/164 [9.8%], p < 0.001). All reconnected PVs were re-isolated.

The 3-month gap in the RL was observed in 13 of 41 patients (31.7%), due to late reconnection (n = 11) or failed index ablation (n = 2). The RL re-ablation led to block in 10 of 13 patients. Late conduction gap in the MIL was found in 15 of 41 patients (36.6%), due to 3-month reconnection (n = 8) or incomplete index ablation (n = 7). After re-ablation, the MIL block was accomplished in 11 of 15 patients.

**Figure 2:**
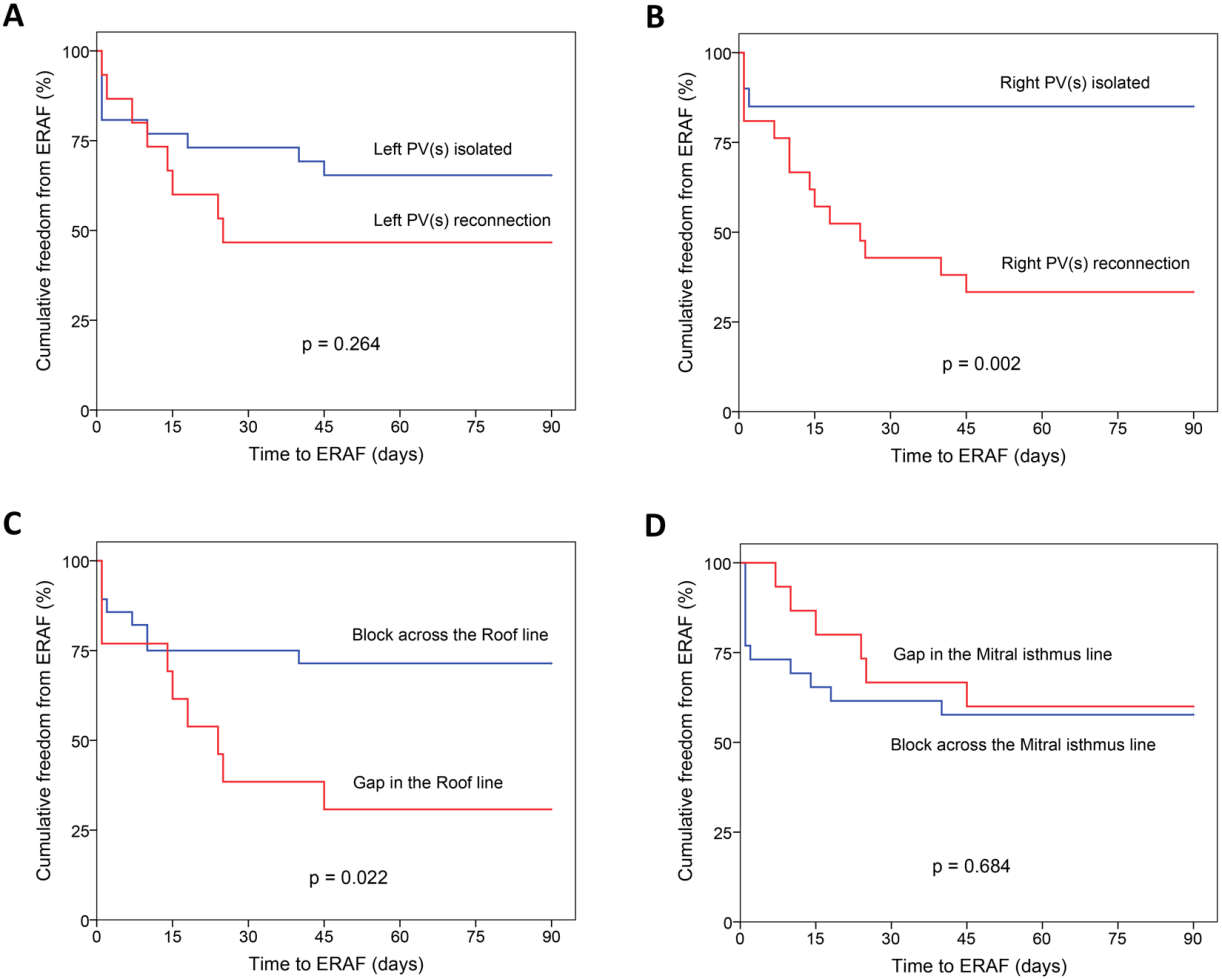
Kaplan-Meier curves of freedom from ERAF during the blanking period after the index ablation in patients grouped by 3-month integrity of the left PVI lesion (**A**), the right PVI lesion (**B**), the Roof line (**C**) and the Mitral isthmus line (**D**).ERAF, early recurrence of atrial fibrillation; PVI, pulmonary vein isolation.

Freedom from ERAF after the index CA procedure was significantly better among patients with than among those without the 3-month integrity of the right PVI lesion (p = 0.002) and the RL (p = 0.022), Fig. 2.

### Risk factors for ERAF

**Figure 3:**
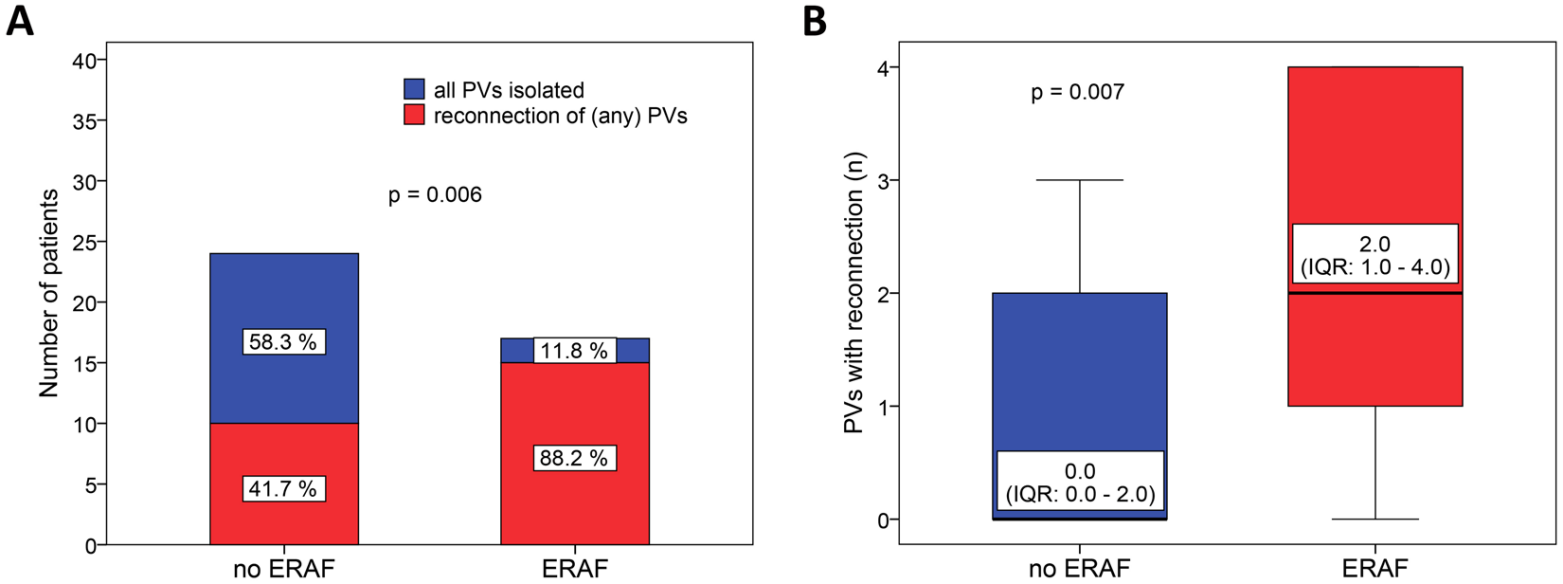
The prevalence (**A**) and the extensiveness (**B**) of PV reconnection at 3 months among patients with and those without ERAF. PV, pulmonary vein; ERAF, early recurrence of atrial fibrillation.

**Figure 4:**
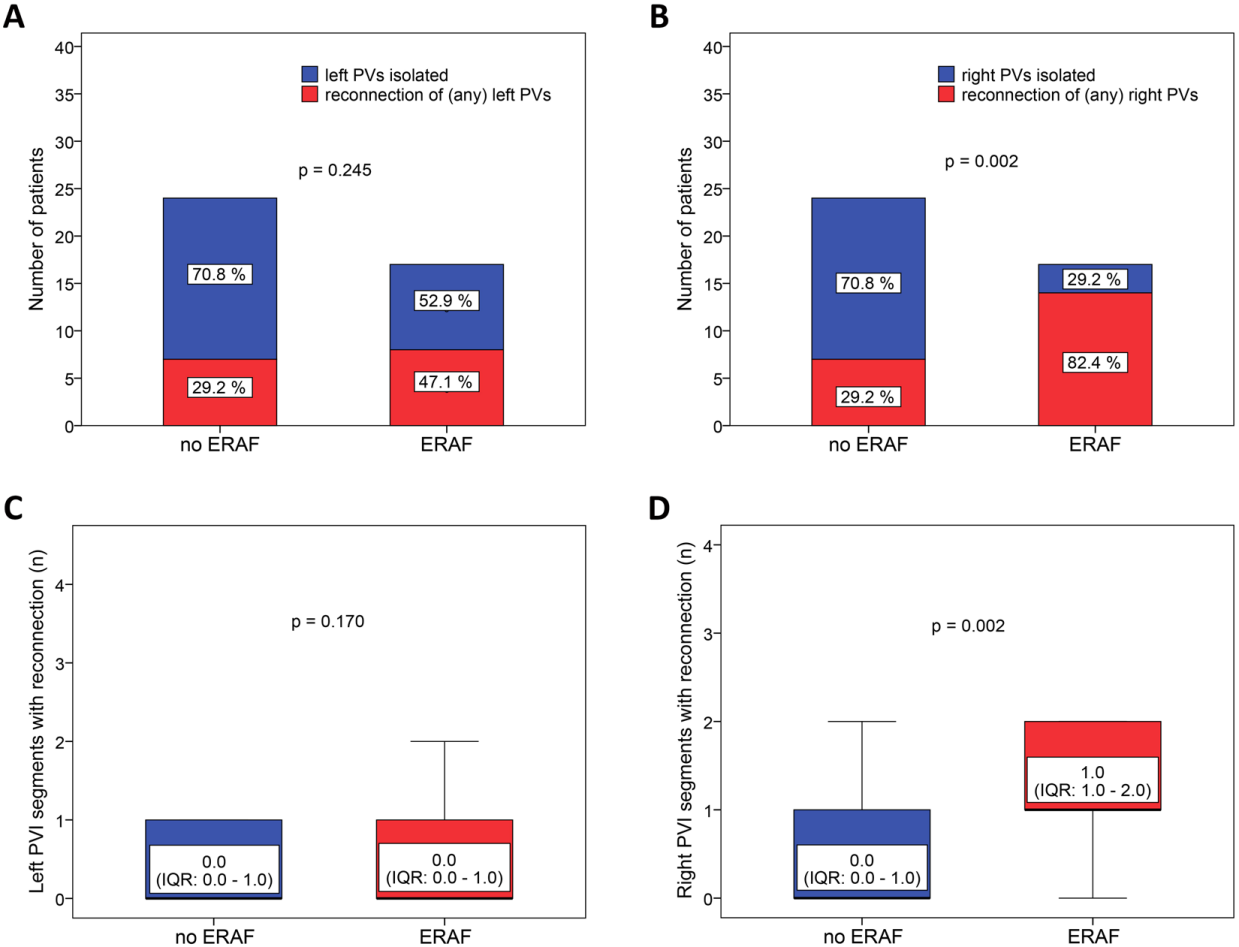
The prevalence (**A**,**B**) and the extensiveness (**C**,**D**) of the 3-month conduction recovery across the left and the right PVI lesions, respectively, in patients categorized based on the ERAF occurrence. PV, pulmonary vein; PVI, pulmonary vein isolation; ERAF, early recurrence of atrial fibrillation.

**Figure 5:**
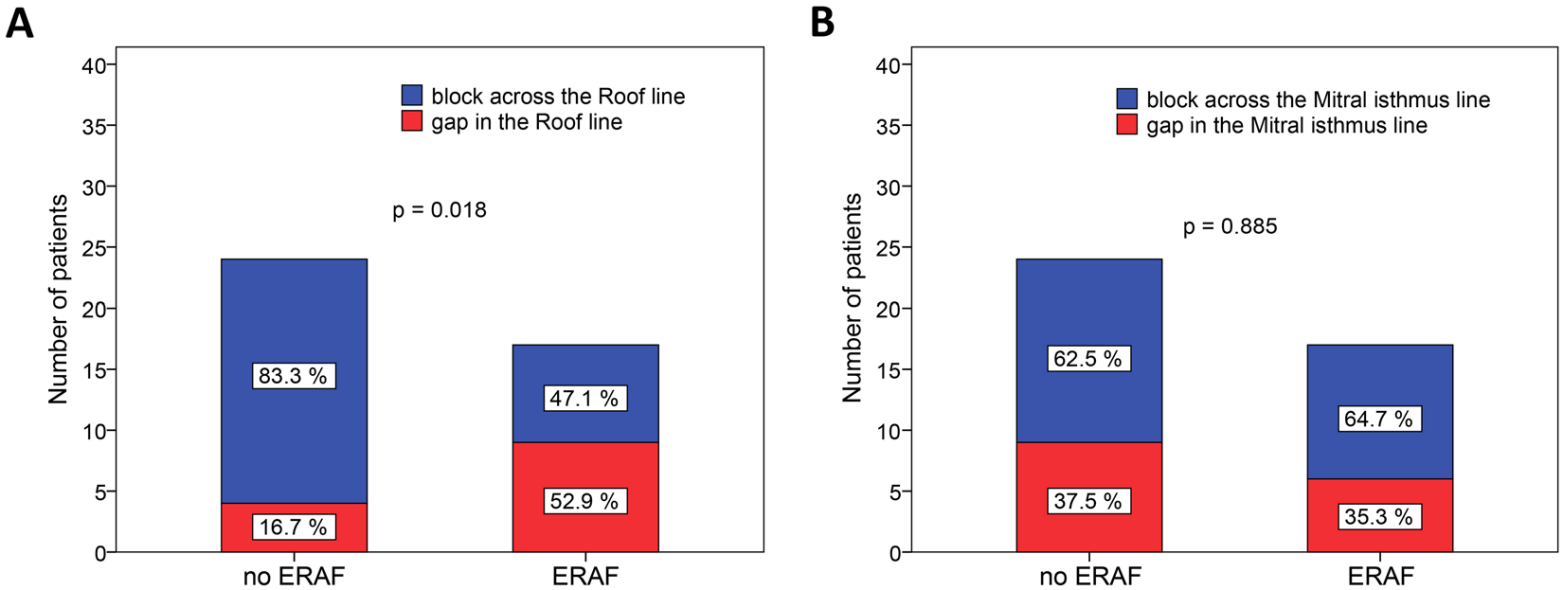
The prevalence of 3-month conduction gap in the Roof line (**A**) and the Mitral isthmus line (**B**) in relation to the ERAF history during the blanking period. ERAF, early recurrence of atrial fibrillation.

Univariate analysis showed that patients with ERAF had significantly longer RF ablation times (p = 0.024) and more reconnections of any PVs (88.2% vs. 41.7%, p = 0.006). Reconnection of the right PVs (82.5% vs. 29.2%, p = 0.002) but not the left PVs (47.1% vs. 29.2%, p = 0.245) at 3 months was significantly higher in those with ERAF compared to without, respectively (Tables 1 and 2, Figs 3,4 and 5). Also, ERAF patients more frequently had a gap in the RL (52.9% vs. 16.7%, p = 0.018) and higher median number of PVs (2.0 [IQR: 1.0–4.0] vs. 0.0 [0.0–2.0], p = 0.007) and right PVI segments (1.0 [1.0–2.0] vs. 0.0 [0.0–1.0], p = 0.002) showing the reconnection. The 3-month gap prevalence in the MIL was similar in patients with and without ERAF (35.3% vs. 37.5%, p = 0.885). No significant difference was identified in the circulatory biomarkers of injury at 24 hours after the index CA, between patients with and without ERAF. Patients with recurrent ERAF episodes and those with single ERAF had similar mean number of reconnected PVs as well as the 3-month gap prevalence in the RL or the MIL (all p > 0.05).

All variables shown by univariate analysis to be significantly associated with ERAF (RF ablation time, reconnection of any PV, reconnection of the right PVs, a gap in the RL, the number of PVs with reconnection, and the number of right PVI segments with reconnection; all p < 0.05) were entered into a multivariate model. However, multivariate analysis identified a significant association only between the number of right PVI segments exhibiting 3-month reconnection and the ERAF occurrence (OR 4.26 [95% CI: 1.57–11.52], p = 0.004).

### Clinical outcome

**Figure 6:**
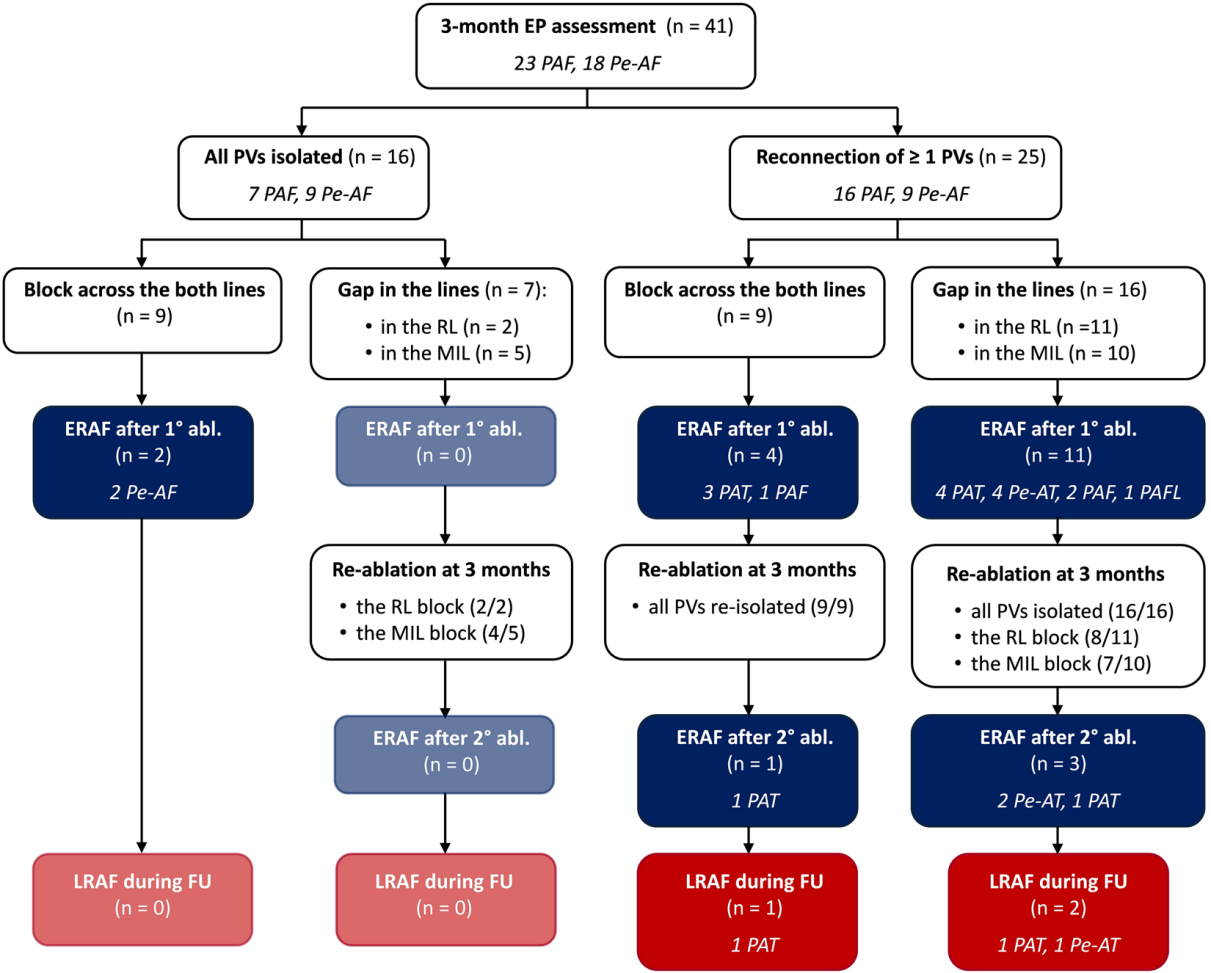
Flow-chart diagram of the clinical course after the index ablation and the 3-month invasive evaluation (with re-ablation). EP, electrophysiology; PAF, paroxysmal atrial fibrillation, Pe-AF, persistent atrial fibrillation; PV, pulmonary vein; RL, roof line; MIL, mitral isthmus line; ERAF, early recurrence of atrial fibrillation; PAT, paroxysmal atrial tachycardia; Pe-AT, persistent atrial tachycardia; LRAF, late recurrence of atrial fibrillation; FU, follow-up.

The arrhythmia outcome is presented in Fig. 6. After the index ablation, ERAF was detected in only 2 of 16 patients (12.5%) with all PVs isolated at 3 months and afterwards these patients were arrhythmia-free off-AADs during the further study course. Majority of patients with ERAF (15 of 17, 88.2%) exhibited 3-month PV reconnection and underwent re-ablation. After the repeated ablation, ERAF was recorded in 4 patients (AT in all). Of these, 3 patients already suffered from ERAF following the index CA procedure. During the median follow-up of 18 months (IQR: 16–19 months) after the last performed CA, the late arrhythmia recurrence was documented in total of 3 patients (AT in all), all of which experienced ERAF after the 3-month ablation procedure. At last follow-up, 38 of 41 patients (92.7%) were arrhythmia-free.

## Discussion

In our study of 41 consecutive AF patients who underwent the uniform set of RF ablation lesions (PVI + RL + MIL) and the follow-up invasive 3-month electrophysiological study, the ERAF occurrence was significantly related to reconnection of the ablation lesions, and not the biomarkers of injury, thus suggesting that the 3-month “blanking” period might not be justified in paroxysmal/persistent AF patients with ERAF post-ablation comprising PVI with additional linear and substrate ablation. However our study was not powered to identify the ERAF patients who would benefit from early re-ablation.

The 3-month ERAF incidence under AAD therapy was 41.2% in our study. In this setting, the 3-month follow-up invasive assessment showed a signficiant association with right PVs reconnection and conduction gaps in the RL among patients with ERAF. The timing and repetitiveness of ERAF was not related to the long-term integrity of the ablation lesion set.

### Clinical significance of ERAF

The reported incidence of ERAF varies considerably, between 16% and 67%, with a pooled estimate of 37.8%^1^. The rate of ERAF is the highest in the first two weeks after CA and then progressively declines^1–3,12^. Our study confirmed these findings.

In some patients, the ERAF is only a temporary event due to the LA inflammation, gradually subsiding over the next weeks^1–3,12^. However as evidenced in this study, the ERAF can be related to the recovery of conduction across the ablation lesion(s) from the index ablation and recent clinical studies identified ERAF as one of the strongest predictors for the late arrhythmia recurrence^2–5^. Currently, data on the relationship between ERAF and the long-term integrity of ablation lesions are mostly limited to patients with clinically overt late arrhythmia relapse which prompted repeat invasive study^2–4^. Only one study of patients with paroxysmal AF reported on the association between the ERAF and durability of PVI^5^. In our study, probably due to the LA linear ablation, two-thirds of ERAFs were organized ATs and not AF. Recurrent ATs after AF ablation are commonly persistent (and not paroxysmal); compared with AF, post-procedural ATs are more commonly associated with hemodynamic deterioration (e.g., heart failure, syncope) due to fast ventricular response and refractoriness to rate-controlling drugs, AADs and cardioversions, thus often requiring subsequent multiple ablation procedures^13^.

### ERAF and the durability of PVI

In the recent study, a protocol mandated invasive remapping 2 months post-PVI showed that the ERAF limited to the first month post-procedure was not related to the late PV reconnection while the ERAF occurring or continuing beyond the first month was strongly associated with more extensive (>1 PV) reconnection^5^. Recent clinical studies supported these findings and demonstrated that the delayed occurrence of ERAF within the 3-month blanking period significantly increased the risk for the late arrhythmia recurrence; nonetheless controversies regarding mechanisms and significance persist^14,15^.

In our study, ERAF was related to the presence as well as the extent of PV reconnection at the 3-month invasive electrophysiological study, without a temporal relationship between the ERAF occurrence and the PVI integrity. Discrepancy between our results and the previous findings could be explained by non-uniform follow-up strategies throughout the blanking period, potentially influencing the ERAF expression and detection^5^. In the previously mentioned study, AAD use was limited to 1 month post-ablation while our patients continued taking AADs for 3 months^5^. In addition, the methods for ERAF screening were different (a handheld ECG-monitoring device vs. 24-hour Holter-monitor). Nevertheless, the ERAF rates in both studies were very similar (17/40 vs. 17/41)^5^. Finally, our study included a discrete ablation strategy that comprised PVI with additional linear ablation that likely influenced temporal healing as well as patients with persistent AF.

In the study by Das *et al*., no discernible relationship between the reconnected PV(s) (i.e., left PVs or right PVs) and the ERAF occurrence was observed^5^. Although the 3-month reconnection rates of the left and right PVs were similar in our study, we found a significantly more reconnections along the right PVI line (vs. left PVI line), mostly across its anterior and posterior segments, correlating with the local atrial wall thickness^16,17^. Although rates of reconnection along the left PVI lines were higher in the ERAF group, the difference was not significant. In addition, a higher number of the right PVI segments with reconnection were observed in those with ERAF compared to those without. Therefore, we speculate that the clinically overt ERAF requires the reconnection of a critical number of fibers at the level of PV-LA junction. Presently, it is not known if a more durable right PVs isolation will positively impact ERAF expression and long-term outcomes.

The following findings of our study merit consideration: first, at the 3-month follow-up study we identified the PV reconnection in almost all patients experiencing ERAF (15 of 17, 88.2%). Since the 3-month procedure included a mandatory re-ablation of the gaps, the long-term clinical significance of these ERAF cases remained unclear. However, according to these findings, probably all patients with ERAF, irrespective to its timing, deserve intense monitoring in the further post-procedural course until the final decision on repeat CA procedure is made. Second, 2 of 16 patients with durable PVI at 3 months had ERAF during the blanking period and afterwards were arrhythmia-free. This finding confirmed that ERAF can be provoked by transient pro-arrhythmic factors operating immediately post-ablation such as inflammation, but the incidence of these “purely temporal” ERAF cases was considerably lower (12.5%) than in the other studies (30–50%)^1–3,14,15^. These findings underline the notion that PVI integrity is one of the most important prerequisites for long-term AF ablation success and highlights the necessity for additional technological and procedural improvements in order to provide a more durable PVI.

### ERAF and the integrity of the Roof line

To the best of our knowledge, we were the first to report the relationship between ERAF and the long-term completeness of the LA lines in a population of patients with paroxysmal and persistent AF. It has been shown that an extensive substrate-based RF ablation (beyond PVI) increased the proportion of patients with transient ERAF due to acute reversible electrophysiological alterations after the procedure^1,6,12^. Contrary to other reports^1–3,12,18^, the levels of biomarkers of inflammation and myocardial necrosis obtained 24 h after CA procedure in our study were similar in patients with and without ERAF, although RF delivery time was significantly longer in those with ERAF probably reflecting the difficulties in reaching the endpoint(s). We showed that incompleteness of the RL (but not MIL) at 3 months was strongly associated with ERAF. The role of the LA roof in the mechanisms of AF has been previously described^10,19,20^. The achievement of RL block resulted in cycle length prolongation, termination and non-inducibility of paroxysmal AF and interruption of the LA reactivation circuits in persistent AF^10,19^. The relative importance of integrity of the RL compared to MIL for ERAF occurrence in our report may be explained by the different distribution of the low-voltage areas reflective of arrhythmogenic substrate at the roof (49%) compared to the mitral isthmus (6%) in AF patients^20^. The adjuvant substrate modification based on these low-voltage areas ablation (beyond PVI) significantly improved the long-term outcome in both patients with persistent and paroxysmal AF^20^. Therefore, we assume that despite the (re)activation of the triggers post-ablation (due to LA inflammation and/or late PV reconnections), the presence of an intact RL block after the index ablation might preclude the perpetuation of clinically detectable ERAF episodes.

### Study limitations

This was a single centre study with a limited number of participants due to the ethical issues of exposing the arrhythmia-free patients to a repeated invasive procedure. The follow-up invasive study included the gap(s) reablation, thus precluding the assessment of long-term clinical significance of ERAF in these patients. However, avoidance of closing the conduction gaps during the redo procedure would have been ethically problematic.

The intermittent rhythm monitoring during the post-ablation follow-up (i.e., 24-hour Holter-monitoring) could affect the ERAF detection^12^ but our strategy was in line with usual clinical practice and current guidelines^1–3^.

We did not use the contact-force catheters that may have potentially compromised the PVI durability. However, the late PV reconnection rate of 62% after contact-force guided PVI in a recent report^5^ as well as the 3-month reconnection distribution and extensiveness along the right PVI line (vs. left PVI line) after PVI performed with contact-force guidance in other studies^17,21^ were similar to our findings.

## Conclusions

In the present study systematically evaluating the relation between ERAF and the index ablation lesion integrity, the incidence of ERAF was 41% during the 3-month follow-up and in most patients ERAF occurred during the first month post-ablation. At the 3-month invasive remapping, patients with ERAF more frequently exhibited any PVs reconnection, right PVs reconnection and a gap in the RL, as well as a more extensive PV reconnection in terms of the higher number of the PVs segments recovering the conduction compared with non-ERAF patients. Our findings question the validity of the “blanking” period approach to determine procedural success in patients that undergo PVI and linear ablation and the assumption that ERAF consistently reflects reversible mechanisms of arrhythmia.
